# Nutritional profile of Syrian refugee children before resettlement

**DOI:** 10.1186/s13031-019-0208-y

**Published:** 2019-06-04

**Authors:** Sweetmavourneen Pernitez-Agan, Kolitha Wickramage, Catherine Yen, Elizabeth Dawson-Hahn, Tarissa Mitchell, Dominik Zenner

**Affiliations:** 1 Migration Health Division, International Organization for Migration, Manila, Philippines; 2 Migration Health Division, International Organization for Migration, Washington, DC USA; 3 Department of Pediatrics, University of Washington, Seattle, WA USA; 4 Seattle Children’s Research Institute, Seattle, WA USA; 5 Division of Global Migration and Quarantine, US Centers for Disease Control and Prevention, Atlanta, GA USA; 6 Migration Health Division, International Organization for Migration, Brussels, Belgium

**Keywords:** Syria, Refugees, Nutrition, Malnutrition, Child health, Obesity

## Abstract

**Background:**

The year 2015 marked the highest number of refugees globally and included record numbers of Syrians moving to neighboring countries. Half of the Syrians were children aged ≤18 years. Our study sought to examine undernutrition and overnutrition among a group of Syrian refugee children who underwent medical screening by IOM for resettlement.

**Methods:**

This is a retrospective review of Syrian refugee children aged 6 to 59 months from January 1, 2015 to December 31, 2016. The World Health Organization (WHO) Stata package computed Z-scores based on available weight and height data. Prevalence estimates of undernutrition (wasting and stunting) and overnutrition (overweight and obesity) were made using WHO standards. Multivariate analysis was used to determine the factors associated with wasting, stunting, and overnutrition, adjusting for age, sex, family size, and country of health assessment.

**Results:**

A total of 14,552 Syrian refugee children aged 6 to 59 months underwent health assessments in Jordan (43·1%), Lebanon (38·8%), Turkey (7·0%), Greece (6·7%), Egypt (2·4%), and Iraq (2·1%). Overall, this group of Syrian refugee children had a low prevalence of wasting (< 5%) and stunting (< 10%), and high prevalence of overweight or obese (10.6%). Differences were observed in the prevalence of wasting by country of health assessment. In the multiple regression analysis, the prevalence of stunting and overnutrition decreased with increasing age, and being male was associated with overnutrition but not wasting and stunting.

**Conclusions:**

Findings revealed an overall low prevalence of undernutrition among this group of Syrian children assessed, although prevalence varied by age group. This low prevalence may reflect the effectiveness, as well as expose possible gaps, of refugee nutrition programs or interventions in countries of asylum. Further studies are recommended to evaluate other possible contributors to malnutrition in this refugee group.

## Background

Malnutrition is a major public health concern in humanitarian emergencies. [1] Malnutrition includes wasting, stunting, underweight, and overnutrition (overweight or obesity). [2] Wasting indicates in most cases a severe process of weight loss, which is often associated with acute shortage of food and/or severe disease. However, wasting may also be the result of a chronic unfavourable condition. Stunting, or low height-for-age, [3] can result from prolonged or repeated undernutrition or gastrointestinal illness [4] and is a key indicator of chronic malnutrition. [3] Chronic malnutrition can lead to irreversible impairment of a child’s cognitive and physical development. Overweight or obesity in childhood is associated with a high risk of obesity in adulthood, which can lead to chronic conditions such as diabetes and cardiovascular disease. [5] People in humanitarian crisis, such as refugees, are at higher risk for malnutrition because they often lack access to sufficient healthy food, opportunities for physical activity, and health services. [6] Refugees are persons who have been forced to leave their country due to persecution, war or violence. [7] This forced displacement leads to multiple health disparities such as access to food and therefore proper nutrition and disruption of vaccination, especially for children. [5]

Conflict in the Syrian Arab Republic, which started in 2011, displaced 4·8 million people as refugees to neighboring countries, such as Turkey, Lebanon, Jordan, Iraq, and Egypt, by the end of 2016. [7] Half of these refugees were children under the age of 18 years. Following this large influx, humanitarian agencies and the governments of host countries had difficulty meeting the growing needs of the refugees, including health services. [8–11] In Iraq, Jordan, and Turkey, many Syrian refugees rented or lived in makeshift dwellings, with only a small proportion (~ 10%) staying in refugee camps. [7] Additionally, there are no official refugee camps in Lebanon, Greece, and Egypt. Recent studies in Lebanon and Jordan have indicated that most Syrian refugees faced some food insecurity, with larger families more vulnerable. [12, 13]

The International Organization for Migration (IOM)--the UN Migration Agency-- together with governments, the United Nations Health High Commissioner for Refugees (UNHCR), non-government organizations and other partners work together in the resettlement process of refugees. The UNHCR endorses refugee cases to governments for resettlement consideration. Once this occurs, IOM, under cooperative agreements with these governments, performs comprehensive health assessments of refugees prior to their resettlement to the receiving country. Components of the health assessment include a general assessment (medical history and physical examination, which includes anthropometric measurements for which IOM nursing staff have been trained to measure using recommended tools and methods), screening tests for specific conditions (e.g. tuberculosis, hepatitis, and others, depending on receiving country requirements), treatment and specialist referrals, assessment for travel health assistance during transportation, and others depending on the government protocols. Health assessment aims to promote the health of assisted refugees and protect the health security of receiving countries.

Our study sought to determine the factors related to wasting, stunting, and overnutrition of a group of Syrian refugee children undergoing health assessments conducted by IOM medical staff prior to resettlement as part of programmes of governments assisted by IOM. Information on the nutritional status of Syrian refugee children can guide health providers in refugee settings and resettlement countries on preparations for receiving Syrian refugee families.

## Methods

### Data source and study population

This study is a retrospective review of data from routine health assessments of Syrian refugee children aged 6 to 59 months conducted by IOM. Health assessment data of all IOM assisted refugees are entered by the attending physician or nurse in the “Migrant Management Operational System Application” (MiMOSA), an IOM data management software. MiMOSA data from IOM health clinics worldwide are aggregated and undergo quality control in a Central Data Repository (CDR) used for generating statistical reports. IOM perform regular validation of health assessment data using automated rules or checks to verify and correct inconsistencies in coordination with the focal persons at the country level operations. Continuous improvements in data quality checking and validation is being developed by IOM. MiMOSA data of Syrian refugee children aged 6 to 59 months for January 1, 2015 through December 31, 2016 were extracted and analyzed. Infants under 6 months were excluded in our study on the assumption that this group is unique in terms of their nutrition and developmental needs. [5] We examined country of health assessment, nationality, age, sex, height, weight, camp or non-camp setting, and family size, which is the number of individuals who are related and travelling together.

### Anthropometric measurements

Measurement of height (or length) and weight followed standard procedures. [14] Trained IOM medical personnel measured the weight of each child using a digital weighing scale or a regular weighing scale. A child’s length was measured lying down and height measured standing up with a measuring tape or a height device attached to the weighing scale.

### Statistical analyses

Data processing and analyses to generate estimates of nutritional indicators used Stata (SE 14.0, Stata Corp). We excluded duplicate entries, repeat health assessments, and unknown height and weight values from our data analyses. In our study, we covered the initial health assessment of children extracted based on the first date of health assessment of a child in 2015 and 2016.

Based on World Health Organization (WHO) guidelines for children under 5 years old, wasting was assessed using weight-for-length or weight-for-height Z-scores (zWFH), while stunting was assessed using length-for-age or height-for-age Z-score (zHFA). Z-scores for anthropometric measurements (zWFH and zHFA) were computed using the WHO Stata package [15] and the 2006 WHO Child Growth Standards. The z-scores were further stratified by severity (Table 1). [3, 16] The WHO standards are based on the growth of a population of children from six sites globally under optimal environmental conditions. [1, 3, 14]

**Table 1:** WHO classification of nutritional status

Nutritional status	Z-score range
Wasting (acute malnutrition)	
*Moderate*	zWFH < −2 and ≥ − 3
*Severe*	zWFH < − 3
Stunting (chronic malnutrition)	
*Moderate*	zHFA < −2 and ≥ −3
*Severe*	zHFA < −3
Healthy Weight	zWFH <=2 and > = − 2
Overweight	zWFH > + 2 and < = + 3
Obesity	zWFH > + 3

*Source:* WHO Global Database on Child Growth and Malnutrition. Available from: http://www.who.int/nutgrowthdb/about/introduction/en/index5.html

WHO classifies the public health significance of acute and chronic malnutrition using prevalence thresholds. We defined undernutrition (wasting and stunting) and overnutrition (overweight and obesity) based on WHO definitions. In the context of emergencies, WHO previously classified prevalence of wasting of less than 5% as low and a prevalence of 10% or greater as high. In stunting, less than 20% is considered low, whereas prevalence of 30% or greater is high (Table 2). [3, 11, 17] Historically, overweight and obesity have not been included in estimates of malnutrition in humanitarian settings; however, increasing evidence shows that both undernutrition and overnutrition can be present. [17–19] A recent study by de Onis et al. (2018), recommended new prevalence thresholds that were developed based on degrees of deviation from normality as defined by the WHO Child Growth Standards. The prevalence thresholds for wasting are the same with previous guidelines with an additional classification of ‘very low’ for prevalence of less than 2.5%. Also, latest classification included overweight thresholds which were the same as for wasting (low is considered a prevalence of < 5%). As for stunting, prevalence of less than 10% were considered low compared to the previous guidelines (< 20%) with very low thresholds at less than 2.5%. A stunting prevalence of 20% or greater were high. [20]

**Table 2:** WHO classification for assessing severity of malnutrition rates by prevalence thresholds

Indicator	Very low	Low	Medium	High	Very High
% Wasting					
Old*	–	< 5 (acceptable)	5–9 (poor)	10–14 (serious)	≥15 (critical)
New**	< 2.5	2.5–5	5- < 10	10- < 15	≥15
% Stuntinga					
Old*	–	< 20 (acceptable)	20–29 (poor)	30–39 (serious)	≥40 (critical)
New**	< 2.5	2.5- < 10	10- < 20	20- < 30	≥30
%Overweight**	< 2.5	2.5–5	5- < 10	10- < 15	≥15

Sources:* WHO Nutrition Landscape Information System (NLIS): Interpretation Guide (2010), p2 and Module 10, p19. Available from http://www.who.int/nutrition/nlis_interpretation_guide.pdf; Prudhon C. Module 10 Nutrition information and surveillance systems. In: The harmonized training package: resource material for training on nutrition in emergencies. Version 2. Oxford: Nutrition Works, Emergency Nutrition Network, Global Nutrition Cluster; 2011**.** ** Onis M De, Borghi E, Arimond M, Webb P, Croft T, Saha K, et al. (2018). Prevalence thresholds for wasting, overweight and stunting in children under 5 years. Public Health Nutrition

Per WHO recommendation, we have excluded z-score values (*n* = 360) with the following range: zWFA<− 6 or zWFA> + 5, zHFA <− 6 or zHFA> + 6, and zWFH<− 5 or zWFH> + 5, as well as records from countries that assessed fewer 40 Syrian refugee children combined. The final analysis included records from children examined in six countries─Jordan, Lebanon, Greece, Turkey, Egypt, and Iraq.

Malnutrition prevalence estimates were calculated, and Chi-square testing was used to assess for differences by country. For children with complete information on family size, a multiple logistic regression analysis was used to determine associations with identified independent variables (age group, sex, family size, and country of health assessment) on malnutrition with each of the outcome variables (wasting, stunting, and overnutrition). Dummy variables were created for the independent variables with more than two categories. Also, adjusted odds ratios were calculated to determine associations between independent variables and outcome variables. A *p*-value of < 0.05 was considered statistically significant.

## Results

### Demographic characteristics

From January 1, 2015 through December 31, 2016, a total of 14,552 Syrian refugee children aged 6 to 59 months underwent health assessments in Jordan (43·1%), Lebanon (38·8%), Turkey (7·0%), Greece (6·7%), Egypt (2·4%), and Iraq (2·1%) (Table 3). Of these children, for those with information on residence at time of health assessment (79·2%), < 1% lived in refugee camps. Slightly more than half of the children were male (51·6%), and many were in older age groups of 48 to 59 months of age (*n* = 3506, 24·1%) and 36 to 47 months of age (*n* = 3496, 24%). The majority (~ 99%) of children were in families with 1 to 10 children; of these, 56% were in families with 1–5 children. Fewer than 1% were in families with 11 or more children.

**Table 3:** Demographics of Syrian refugee children aged 6 to 59 months assessed by IOM, 1 January 2015 to 31 December 2016

Characteristic	Jordan (n (%))	Lebanon (n (%))	Turkey (n (%))	Greece (n (%))	Egypt (n (%))	Iraq (n (%))	All (n (%))
Number of children	6267	5645	1020	969	352	299	14,552
Age (mo)							
Mean age (SD)*	34.1 (15.6)	35.1 (15.4)	34.0 (15.5)	32.4 (15.7)	36.0 (15.3)	33.3 (15.4)	34.4 (15.5)
Median age (IQR)**	35.0 (26.8)	35.9 (25.9)	34.4 (26.7)	31.5 (27.6)	38.4 (25.9)	33.3 (25.3)	35.2 (26.5)
Age group (mo)							
6 to 11	613 (9.8)	471 (8.3)	101 (9.9)	100 (10.3)	24 (6.8)	29 (9.7)	1338 (9.2)
12 to 23	1351 (21.6)	1122 (19.9)	217 (21.3)	260 (26.8)	64 (18.2)	67 (22.4)	3081 (21.2)
24 to 35	1311 (20.9)	1234 (21.9)	218 (21.4)	217 (22.4)	77 (21.9)	74 (24.8)	3131 (21.5)
36 to 47	1522 (24.3)	1390 (24.6)	246 (24.1)	186 (19.2)	84 (23.4)	68 (22.7)	3496 (24.0)
48 to 59	1470 (23.5)	1428 (25.3)	238 (23.3)	206 (21.3)	103 (29.3)	61 (20.4)	3506 (24.1)
Sex							
Male	3187 (50.9)	2972 (52.7)	549 (53.8)	481 (49.6)	174 (49.4)	147 (49.2)	7510 (51.6)
Female	3080 (49.2)	2673 (47.4)	471 (46.2)	488 (50.4)	178 (50.6)	152 (50.8)	7042 (48.4)
Family size***							
1 to 5	2663 (50.4)	2680 (60.3)	371 (66.3)	150 (70.4)	142 (60.4)	103 (69.6)	6109 (56.2)
6 to 10	2581 (48.9)	1711 (38.5)	189 (33.8)	62 (29.1)	93 (39.6)	43 (29.1)	4679 (43.0)
11 and up	38 (0.7)	48 (1.1)	0	1 (0.5)	0	2 (1.4)	89 (0.82)
Population type***							
In-camp	54 (1.6)	no camp	24 (2.4)	no camp	no camp	12 (8.8)	90 (0.8)
Out-camp	3343 (98.4)		996 (97.6)			125 (91.2)	11,430 (99.3)

*SD, standard deviation

**IQR, interquartile range

***Data on family size and population type only available for 79.2% (*n* = 11,520) of the sample

### Nutritional status

#### Prevalence of malnutrition

Most children had normal weight-for-length/height (85·6%, *n* = 12,466) and length/height-for-age (85·2%, n = 12,398). Approximately 10·6% (*n* = 1547) of children were either overweight (*n* = 1277) or obese (*n* = 270). The prevalence of wasting was 3·7% (*n* = 539); among these children, 73% (*n* = 395) had moderate wasting, and 27% (*n* = 144) had severe wasting. The prevalence of stunting was 9·1% (*n* = 1331); among these children, 72% (*n* = 958) were moderately stunted, while 28% (*n* = 373) were severely stunted. Less than 1% (0·4%, *n* = 56) of children were both stunted and wasted (data not shown).

**Fig. 1:**
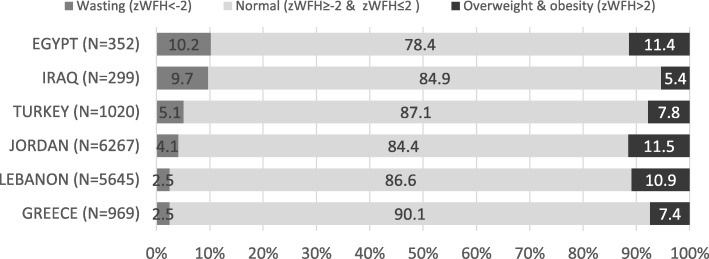
Wasting and overweight/obesity prevalence of Syrian refugee children by country, 1 January 2015 to 31 December 2016 The figure shows the prevalence (%) of wasting (zWFH<− 2), normal weight (zWFH≥ − 2 & zWFH≤2), and overweight and obesity (zWFH> 2) of Syrian refugee children aged 6 to 59 months assessed by IOM. Calculation of prevalence was done by using the number of children that falls under each category of the weight for height Z-score as numerator and the total number of children assessed by country of health assessment as denominator.

Wasting and stunting prevalence significantly differed among the six countries (*p* = .001). The overall prevalence of wasting was 3·7%, with the lowest prevalence observed in Lebanon (2·5, 95% CI, 2·1, 2.9) and Greece (2·5, 95% CI, 1·6, 3·7) and the highest in Egypt (10·2%. 95% CI, 7·3, 13·9) and Iraq (9·7, 95% CI, 6·6, 13.6) (Fig. 1). A low prevalence of stunting was observed across all countries (9·1%), ranging from 7% (95% CI, 4·4, 10·5) and 7.5% (95% CI, 6·9, 8·2) in Iraq and Jordan, respectively, to 14.8% (95% CI, 12·7, 17·1) in Turkey.

The prevalence of overweight and obesity combined was highest in Jordan (11·5, 95% CI, 10·7, 12·3), Egypt (11· 4, 95% CI: 8· 2, 15·, and Lebanon (10·9, 95% CI, 10·1, 11·8), 2) and lowest in Iraq (5·4, 95% CI, 3·9, 8·5), Greece (7·4, 95% CI, 5·9, 9·3), and Turkey (7·8%, 95% CI, 6·3, 9·7).

#### Determinants associated with malnutrition

Approximately 75% (10,877/14,522 children) had complete information on age, sex, and family size. All three models from the multiple regression analysis revealed significant associations of the identified independent variables with wasting, stunting and overnutrition (*p*-value< 0.01).

In the analysis, age was associated with wasting, stunting, and overnutrition (Table 4). The odds of wasting in the 6 to 11 months age group was twice that of the 12 to 23 months age group (OR 2·16, 95% CI: 1·54, 3·03). The odds of stunting and overnutrition was also highest in this age group, but this difference was not statistically significant when compared to those 12 to 23 years. The odds of stunting and overnutrition decreased with increasing age.

**Table 4:** Nutrition status of Syrian refugee children*, prevalence and results of multivariable analysis, IOM, 1 January 2015 to 31 December 2016

		Wasting (zWFH <− 2)***			Stunting (zHFA < − 2)***			Overweight & Obesity (zWFH > 2)***		
	N	% (95% CI)	OR (95% CI)**	*p*-value	% (95% CI)	OR (95% CI)**	*p*-value	% (95% CI)	OR (95% CI)**	*p*-value
All children*	10,877	3.6 (3.2, 3.9)			8.8 (8.3, 9.4)			11.6 (11.0, 12.2)		
Age group										
6 to 11	985	6.8 (5.3, 8.5)	2.09 (1.51, 2.91)	**0.0001**	13.3 (11.2, 15.6)	2.56 (2.00, 3.28)	**0.0001**	17.4 (15.1, 20.0)	2.33 (1.88, 2.90)	**0.0001**
12 to 23	2280	3.3 (2.6, 4.1)	0.97 (0.71, 1.33)	0.8520	12.1 (10.8, 13.5)	2.29 (1.86,2.81)	**0.0001**	15.0 (13.6, 16.5)	1.97 (1.64, 2.36)	**0.0001**
24 to 35	2349	3.1 (2.4, 3.9)	0.92 (0.67, 1.27)	0.6270	8.9 (7.8, 10.2)	1.59 (1.28, 1.97)	**0.0001**	10.6 (9.4, 11.9)	1.33 (1.10, 1.61)	**0.0030**
36 to 47	2639	3.3 (2.7, 4.1)	0.99 (0.73, 1.33)	0.9250	7.1 (6.2, 8.2)	1.24 (0.99, 1.54)	**0.0570**	10.7 (9.5, 11.9)	1.34 (1.11, 1.61)	**0.0020**
48 to 59	2624	3.3 (2.7, 4.1)	Ref.		5.9 (5.1, 6.9)	Ref.		8.2 (7.2, 9.4)	Ref.	
Sex										
Male	5636	3.5 (3.1, 4.0)	Ref.		9.0 (8.2, 9.7)	Ref.		13.0 (12.2, 13.9)	Ref.	
Female	5241	3.6 (3.1, 4.2)	1.03 (0.84, 1.27)	0.7570	8.7 (7.9, 9.5)	0.97 (0.85, 1.11)	0.6240	10.0 (9.2, 10.9)	0.74 (0.66, 0.83)	**0.0001**
Family size										
1 to 5	6109	3.9 (3.4, 4.4)	Ref.		8.1 (7.4, 8.8)	Ref.		12.2 (11.4, 13.1)	Ref.	
6 to 10	4679	3.2 (2.7, 3.7)	0.81 (0.65, 1.00)	0.0460	9.8 (9.0, 10.7)	1.35 (1.17, 1.54)	**0.0001**	10.7 (9.9, 11.7)	0.87 (0.77, 0.99)	0.0310
11 and up	89	4.5 (1.2, 11.1)	1.31 (0.47, 3.64)	0.5990	7.9 (3.2, 15.5)	1.09 (0.50, 2.39)	0.8250	11.2 (5.5, 19.7)	0.95 (0.49, 1.85)	0.8770
Country										
Jordan	5282	4.1 (3.6, 4.7)	Ref.		7.4 (6.7, 8.1)	Ref.		11.9 (11.0, 12.8)	Ref.	
Lebanon	4439	2.4 (1.9, 2.9)	0.56 (0.44,0.71)	**0.0001**	9.6 (8.7, 10.5)	1.40 (1.21, 1.61)	**0.0001**	11.9 (11.0, 13.0)	1.00 (0.88, 1.13)	0.9780
Turkey	560	4.6 (3.1, 6.7)	1.10 (0.72, 1.67)	0.6640	13.2 (10.5, 16.3)	2.00 (1.53, 2.62)	**0.0001**	10.0 (7.6, 12.8)	0.79 (0.59, 1.06)	0.1140
Egypt	235	8.1 (4.9, 12.3)	2.09 (1.28, 3.41)	**0.0030**	16.6 (12.1, 22.0)	2.73 (1.90, 3.93)	**0.0001**	9.4 (5.9, 13.8)	0.79 (0.50, 1.24)	0.3030
Greece	213	3.2 (1.3, 6.7)	0.75 (0.35, 1.63)	0.4730	7.5 (4.4, 11.9)	1.04 (0.62, 1.75)	0.8850	6.6 (3.6, 10.8)	0.50 (0.29, 0.86)	**0.0130**
Iraq	148	9.5 (5.3, 15.4)	2.38 (1.35, 4.21)	**0.0030**	7.4 (3.8, 12.9)	1.06 (0.57, 1.98)	0.8560	6.1 (2.8, 11.2)	0.47 (0.24, 0.92)	**0.0280**

*Considered only the six top countries with information on family size

**Odds ratios were adjusted for the other variables

***All models of each outcome variable were significant (*p*-value< 0.01)

On analysis adjusted for age, family size, and country of health assessment, male sex was associated with overnutrition (OR 1·35, 95% CI: 1·20, 1·52) but not with wasting or stunting. The odds of wasting were highest among children from families of 11 or more members (OR 1·31, 95% CI: 0·47, 3·64) compared with families of 1 to 5 members; however, the association was not statistically significant. For stunting, a significant association was found among children from families of 6 to 10 members (OR 1·35, 95% CI: 1·17, 1·54).

## Discussion

The overall prevalence of wasting (3·7%) among Syrian refugee children in this assessment was low as defined by WHO prevalence thresholds. Our findings were consistent with results of surveys done in 2012 to 2016 in selected locations (Table 5). In terms of stunting prevalence, our study showed lower prevalence (9·1%) compared with most of the previous surveys. However, years before the crisis, children in the Syrian Arab Republic had high wasting prevalence (11.1% in 2001, 10% in 2006 and 2009) and stunting prevalence (31·1% in 2001, 28·6% in 2006, and 27·5% in 2009). [21] Hossain et al. suggested that the nutritional status of Syrian refugee children is comparable with that of the host communities. [19] Factors that may influence such nutritional outcomes include food supply (at camp or host community households), cultural practices (e.g., breastfeeding), potential comorbidities (eg, helminthic infections), and work restrictions (e.g., work permits).

**Table 5:** Nutrition status of Syrian children in Jordan, Lebanon, and Iraq from other studies/reports

Country	Location/ setting	Author (Reference no.)	Year of survey	Wasting		Stunting		Overweight		Age (mo)
				N	% (95% CI)	N	% (95% CI)	N	% (95% CI)	
Jordan	Overall	UNICEF [22]	2014	804	1.0	779	13.0	804	2.8	6 to 59
		Hossain [24]	2014	896	1.0	896	13.6	–	–	< 60
		UNHCR [23]	2016	1282	2.1	1268	12.3	1282	1.4	6 to 59
	Zaatari camp	UNICEF [22]	2014	325	1.2 (0.5, 3.2)	312	17.0 (11.7, 24.0)	325	3.1 (1.6, 5.7)	6 to 59
		Hossain [24]	2014	355	1.2 (0.5, 3.2)	355	16.7 (11.6, 23.4)	–	–	< 60
		UNHCR [23]	2016	377	2.7 (1.4, 5.0)	373	11.3 (8.5, 15.2)	377	1.6 (0.7, 3.4)	6 to 59
	Azraq camp	UNHCR [23]	2016	418	1.9 (0.9, 4.2)	411	19.2 (16.0, 22.9)	418	1.0 (0.4, 2.5)	6 to 59
	Out-camp	UNICEF [22]	2014	479	0.8 (0.3, 2.2)	467	9.0 (6.5, 12.3)	479	2.5 (1.4, 4.5)	6 to 59
		Hossain [24]	2014	541	0.8 (0.3, 2.2)	541	10.5 (7.6, 14.4)	–	–	< 60
		UNHCR	2016	487	1.8 (1.0, 3.4)	484	6.4 (4.4, 9.3)	487	1.6 (0.8, 3.2)	6 to 59
Lebanon	Overall	UNICEF [26]	2013	1384	5.9 (4.8, 7.1)	1384	17.3 (15.1, 19.7)	–	–	6 to 59
		Hossain [24]	2013	1535	2.2	1535	19.1	–	–	< 60
	North	UNICEF [26]	2013	360	6.7 (4.6, 9.5)	360	17.2 (13.3, 22.1)	–	–	6 to 59
		Hossain [24]	2013	409	3.9 (2.5, 6.1)	409	20.1 (15.6, 25.4)	–	–	< 60
	South	UNICEF [26]	2013	302	4.3 (2.7, 6.9)	302	19.9 (15.8, 24.6)	–	–	6 to 59
		Hossain [24]	2013	329	0.3 (0.0, 2.6)	329	21.1 (16.6, 26.2)	–	–	< 60
	Beirut	UNICEF [26]	2013	419	4.1 (2.6, 6.4)	419	13.4 (10.4, 17.0)	–	–	6 to 59
		Hossain [24]	2013	465	0.7 (0.2, 2.3)	465	14.1 (10.8, 18.2)	–	–	< 60
	Bekaa	UNICEF [26]	2013	303	8.9 (5.7, 13.7)	303	20.1 (15.1, 26.3)	–	–	6 to 59
		Hossain [24]	2013	332	4.4 (2.3, 8.2)	332	21.0 (16.0, 27.2)	–	–	< 60
Iraq	Domiz camp	Hossain [24]	2013	944	4.1 (2.8, 6.1)	944	19.0 (15.58, 22.7)	–	–	< 60
	Al-Qa’im	MoH Iraq [27]	2012	589	4.8	589	15.1	–	–	6 to 59

The low prevalence of undernutrition in our study could be due to effective refugee assistance programs or better population-level, pre-crisis nutritional status. [9, 22] It is important to note that majority of Syrian refugees are spread out within the host communities. [7] Aside from difference in living conditions, refugee families living in camps may have more employment restrictions which may affect their purchasing power compared to those residing in the host communities. Surveys conducted in Jordan in 2014 and 2016 reported a low prevalence of wasting (average range: 1·0% to 2·1%) and a higher stunting prevalence (range: 12·3% to 13·6%), regardless of whether the child lived inside or outside a camp. [23–25] While surveys conducted in 2013 in Lebanon showed variable wasting prevalence (2·2% and 5·9%), the stunting prevalence (17·3% and 21·1%) was closer to our observations. [24, 26] In Iraq, wasting prevalence was reported to be low in previous years (4·1% and 4·8% in 2013 and 2012, respectively). [24, 27]

Regarding age, our study found that wasting prevalence was highest (6·8%) among children aged 6 to 11 months. This finding may be related to weaning and breastfeeding practices. [12, 25] For example, a UNICEF survey conducted in Jordan in 2014 showed late introduction of solid and semi-solid food among children aged 6 to 8 months and a low prevalence of exclusive breastfeeding among children aged ≤5 months. [28] Also, food availability may determine the weaning practices of Syrian mothers.

Further, our study revealed that the overall prevalence of overnutrition was 11%, with high prevalence levels found among children in Jordan (11.5%) and Lebanon (10.9%). This could be due partly to the poor quality and variety of food provided to the children. Although the dietary diversity of surveyed Syrian refugees in Jordan was considered acceptable, the consumption of vitamin A and iron rich food were considered low. Majority of refugees surveyed both in-camp and out-camp consumed cereals, spices, condiments and beverages, oils and fats, sweetened food, and vegetables. [23] In Lebanon, Syrian refugee households mainly consumed breads and pasta, condiments, spices, sugar and fats, with limited or no consumption (> 70%) of animal proteins and vitamin A rich fruit and vegetables in their weekly diet. [12] Food choices is complex due to the many cross-cutting conditions (e.g. political setting, access and availability of humanitarian assistance, and, social and cultural beliefs) that affect an individuals’ priority. It is interesting to note that in 2010, WHO identified Egypt, Jordan, Lebanon, and the Syrian Arab Republic as countries in early nutrition transition, described as countries with moderate levels of overweight and obesity, moderate levels of undernutrition in specific population and age groups, and widespread nutrition deficiencies. [29]

A strong predictor of poverty in crisis situations is large family size, as evidenced in a study of Syrian families in Jordan, where the average family size is 7 members. [8, 9] In our study, however, over half the children belonged to smaller families of 1 to 5 members, and family size was not significantly associated with wasting. Still, odds of stunting were significantly higher for children belonging to families of 6 to 10 members than for those in families of 1 to 5 members (OR 1·28, 95% CI: 1·12, 1·47). Children born in larger families may be at greater risk of stunting because of closely spaced child births, maternal malnutrition contributing to low birth weight, or food insecurity. [4, 30]

The differences in nutritional status by country of health assessment may have various causes. The higher prevalence of wasting (10·2%) in refugee children in Egypt could relate to the shift to targeted assistance (based on poverty or economic vulnerability) implemented by the World Food Program and the United Nations High Commissioner for Refugees (UNHCR) by mid-2015, which reduced the number of beneficiaries by half. [31] Between 5 and 10% of children in Iraq and Turkey were also noted to have wasting. Based on a 2016 report, 61·1% (139,360/227,971) of Syrian refugees in Iraq lived within host communities, the majority in the Kurdistan Region of Iraq alongside many Iraqis displaced by the Islamic State of Iraq and the Levant (ISIL) crisis. [7, 32] Despite overcrowding and competition with other refugee populations for resources, some camp-based refugees in Iraq have access to livelihood opportunities. [7] Turkey hosts the largest number of Syrian refugees (> 2 million by the end of 2016). [7] Almost all of these live in rented apartments or houses, with few in camps along the Turkish-Syrian borders. Syrian families in Turkey encounter challenges like those in Iraq, with the addition of language barriers. [33] In 2016, IOM initiated health assessments of Syrian refugees in Greece housed in apartments funded by UNHCR, mainly in Attica. Our study showed that Syrian refugee children in Greece had a low prevalence of wasting (< 5%).

This study has several limitations. First, this is a cross-sectional study, and prevalence estimates were taken from refugee health assessments (as compliance to the resettlement programmes of receiving country governments) conducted in specific settings and time periods, limiting generalizability to other Syrian children and refugee groups. Second, we analyzed routinely collected anthropometric data but did not have access to data regarding other risk factors for malnutrition, such as education level of parents, weight status of parents, source of income, access to health services, length of stay in host country, location of origin in Syria, feeding and weaning practices, referral to malnutrition treatment, comorbid health conditions, and burden of gastrointestinal illness. Third, health assessment of assisted refugees is mainly used as a general screening tool for resettlement depending on the requirements set by governments before departure of refugees to country of destination. Fourth, missing height and weight values are missed cases and may affect the analysis. Also, there is a possibility that the attending medical staff did not enter the measurements). Lastly, our study did not classify children based on refugee status (i.e. asylum seeker or refugee claimant) or existing health conditions (e.g. mental health issues, developmental delays, etc.).

## Conclusions

Despite the known negative nutritional impact of forced displacement and the high influx of Syrian refugees to the host countries, [9, 34, 35] the nutritional status of the group of Syrian refugee children in this study varied by host country, and found low undernutrition and high overnutrition by WHO standards. The variation between countries may relate to income opportunities available to refugees and/or assistance provided by governments and humanitarian groups. However, malnutrition rates should be interpreted in the context of mortality rates, disease prevalence, seasonality, and underlying contributors (related to food, health interventions, and economic and social factors) in the host countries and settings (in-camp and out-camp). [3, 35]

With the ongoing conflict in the Syrian Arab Republic and increasing refugee efflux, the living conditions of Syrian refugees are expected to worsen. Syrian refugee families are considered at high risk of poverty and food insecurity. [9, 10, 12] Thus, continued support from governments and humanitarian organizations is crucial. Programs and partnerships that lead to sustainable livelihood or employment could lower the risks of food insecurity and malnutrition. [9, 14] Analyses of IOM’s health assessment data can help inform the decisions of health providers, governments, and other humanitarian partners. Further studies are needed to examine other known predictors of malnutrition (such as socio-demographics, conditions of host countries, child care practices, vaccination coverage, and selected health conditions), to guide development of appropriate interventions to support the nutritional needs of Syrian refugee populations pre- and post-resettlement.

## Data Availability

The dataset used and analyzed during the current study is not publicly available due to sensitivity of information but is available from the corresponding author on reasonable request.
